# *Gryllus bimaculatus* Extract Protects against Lipopolysaccharide-Derived Inflammatory Response in Human Colon Epithelial Caco-2 Cells

**DOI:** 10.3390/insects12100873

**Published:** 2021-09-27

**Authors:** Kyong Kim, Eun-Young Park, Dong-Jae Baek, Se-Eun Jang, Yoon-Sin Oh

**Affiliations:** 1 Department of Food and Nutrition, Eulji University, Seongnam 13135, Korea; kim_kyong@hanmail.net (K.K.); sejang@eulji.ac.kr (S.-E.J.); 2 College of Pharmacy and Natural Medicine Research Institute, Mokpo National University, Mokpo 58554, Korea; parkey@mokpo.ac.kr (E.-Y.P.); dbaek@mokpo.ac.kr (D.-J.B.)

**Keywords:** *Gryllus bimaculatus*, Caco-2, lipopolysaccharides, inflammatory mediators, tight junction

## Abstract

**Simple Summary:**

Inflammatory bowel disease (IBD), a potentially life-threatening disease, is characterized by increased tight junction permeability and overproduction of proinflammatory cytokines. The long-term administration of recognized chemotherapeutic agents can cause serious potential side effects. As such, increasing attention has been paid to natural, low-toxicity products with anti-inflammatory properties for treating IBD. We assessed the potential utility of the edible cricket species *Gryllus bimaculatus* for anti-inflammatory and cytoprotective effects in the human epithelial cell line Caco-2, following treatment with an inflammatory lipopolysaccharide stimulus. We found that aqueous ethanolic *G. bimaculatus* extract (AE-GBE) treatment increased cell viability and significantly reduced inflammatory mediators. Moreover, AE-GBE significantly reduced inflammatory cytokine expression levels, intestinal epithelial permeability, and related tight junction protein expression levels. In conclusion, AE-GBE can protect epithelial cells from lipopolysaccharide-induced impaired barrier integrity by increasing tight junction proteins and preventing various inflammatory mediators. These results may be used to pursue further use of natural insect extracts in treating IBD.

**Abstract:**

Increased tight junction permeability and overproduction of proinflammatory cytokines are crucial pathophysiological mechanisms in inflammatory bowel disease (IBD). This study evaluated anti-inflammatory effects of aqueous ethanolic *Gryllus bimaculatus* extract (AE-GBE) against intestinal permeability on lipopolysaccharide (LPS)-treated Caco-2 cells. Treatment with AE-GBE increased cell viability and significantly reduced inflammatory mediators such as nitric oxide and LPS-induced reactive oxidative stress. LPS increased the expression levels of iNOS, Cox-2, and 4-hydroxylnonenal; however, these levels were attenuated by AE-GBE treatment. Moreover, the mRNA and protein expression levels of the inflammatory cytokines TNFα, IL-6, IL-1β, and IFNγ were increased by LPS, but were significantly reduced by AE-GBE treatment. Intestinal epithelial permeability and the related expression of the proteins Zoula ocludence-1, occludin, and claudin-1 was increased by LPS treatment, and this effect was significantly reduced by AE-GBE treatment. The reduction in AMPK phosphorylation in LPS-treated Caco-2 cells was reversed in activation by co-treatment with AE-GBE. In conclusion, AE-GBE can protect epithelial cells from LPS-induced impaired barrier integrity by increasing tight junction proteins and preventing various inflammatory mediators. Thus, AE-GBE has the potential to improve inflammation-related diseases, including IBD, by inhibiting excessive production of inflammation-inducing mediators.

## 1. Introduction

The intestine is a vital organ that absorbs, processes, and transports nutrients into the body. Ulcerative colitis and Crohn’s disease are two inflammatory bowel diseases (IBD) caused by intestinal dysfunctions [1,2,3]. IBD results from a defective immune system. Whereas a healthy immune system protects the body from attack by external organisms such as viruses and bacteria, immune systems affected by IBD react abnormally to various environmental factors that cause gastrointestinal inflammation. IBD’s aetiology and various therapies are being developed as the number of IBD patients increases worldwide; however, the long-term administration of recognized chemotherapeutic agents such as aspirin and nonsteroidal anti-inflammatory drugs can cause serious potential side effects. Therefore, replacement therapy for IBD is urgently required. As such, increasing attention has been given to natural, low-toxicity products with anti-inflammatory effects.

IBD decreases quality of life and can sometimes lead to life-threatening complications. A major phenomenon of IBD is in downregulating the expression of molecules that are related to intestinal epithelial cell tight junctions (TJs) [4]. The TJ’s physiological function is maintained by three important known proteins: Zoula ocludence-1 (ZO-1), occludin, and claudin-1. Impairment of the TJ barrier contributes to the progress of inflammation through increased intestinal permeability and altered intestinal microflora [5,6,7].

Lipopolysaccharide (LPS), one of the components constituting the outer membrane of gram-negative bacteria, combines to Toll-like receptor 4 expressed in macrophages and increases cytokine secretion to induce a systemic inflammatory response [3,8]. Studies have reported that LPS acts as a serious pathogenic factor, inducing inflammation in intestinal tissue, and elevated plasma LPS levels were shown in Crohn’s disease and necrotizing enteritis with impaired intestinal permeability [9,10]. Therefore, an attempt to improve the intestinal inflammatory response induced by LPS may be a beneficial method for the prevention or treatment of IBD and may contribute to elucidating the pathogenic mechanism.

Adenosine monophosphate-activated kinase (AMPK) is known to be a central regulator of intestinal barrier homeostasis in epithelial proliferation and differentiation. AMPK’s inactive state contributes to many pathologic processes, such as diabetes, tumour formation, aging, and inflammatory disease [11,12]. 

In this study, we tested an extract of the two-point cricket (*Gryllus bimaculatus*) as an eco-friendly new material with the advantage of producing high protein at a lower cost than livestock [13,14,15]. Various pharmaceutical effects of *G. bimaculatus* extract have been reported, including antihypertensive, antipyretic, anti-inflammatory, anti-obesity, antidiabetic, and anti-aging effects [16,17,18,19]. However, research on the pharmacological activity of aqueous ethanolic *G. bimaculatus* extract (AE-GBE) is still limited and further studies are needed in relation to intestinal dysfunction.

The purpose of this study was to evaluate the effect of AE-GBE on improving intestinal dysfunction by acting as an anti-inflammatory agent and inhibiting inflammation-inducing factors, following a lipopolysaccharide (LPS) stimulus against human epithelial Caco-2 cells, which are commonly used as in vitro IBD models. We investigated AMPK’s activation to elucidate the regulatory mechanism of the TJ proteins affecting intestinal permeability.

## 2. Materials and Methods

### 2.1. Preparation of Aqueous Ethanolic Gryllus Bimaculatus Extract (AE-GBE)

Dried *Gryllus bimaculatus* was purchased from Yechun Bugs land (Yecheon-gun, Gyungsangbuk-do, Korea). *Gryllus bimaculatus* was shredded in a blender, and extracted at least three times with 70% ethanol for 3 h at 60 °C. The extract was filtered using filter paper and concentrated with a vacuum rotary evaporator at 40 °C and then freeze-dried. The yield was 8.2% compared with the powdered sample, and AE-GBE was dissolved in deionized water for the experiment. 

### 2.2. Cell Culture and Cell Viability 

Caco-2 cells were grown in 37 °C with 5% CO_2_ in MEM medium containing 25 mM HEPES (Welgene, Daegu, Korea), 10% FBS (Gibco, Paisley, UK), and 1% penicillin-streptomycin antibiotics (welgene). For cell toxicity assay on AE-GBE, the cells were treated with AE-GBE at different concentrations of 50, 100, 250, 500, and 1000 μg/mL for 24 h. Then, MTT (Duchefa Biochemie BV, Haarlem, Netherlands) of 0.5 mg/mL concentration was added to each plate and further cultured for 2 h; after removal of the supernatant, insoluble formazan crystals were dissolved in 2-propanol and measured at 540 nm (TECAN Group Ltd., Shanghai, China). As another method to analyse cell viability, ATP levels in the cells were measured with the Perkin-Elmer ATPLite system (PerkinElmer Life Sciences, Boston, MA, USA), according to the manufacturer’s instructions [20]. This system was based on the production of light caused by the reaction of ATP with added luciferase and D-luciferin. The attached mammalian cell lysis solution releases the adenine nucleotides and inactivates endogenous ATP degrading enzymes.

### 2.3. Mesurement of Intracellular ROS 

Intracellular reactive oxygen species (ROS) in LPS (1 μg mL^−1^)-induced Caco-2 cells for 48 h were measured using 2′,7′-dichlorofluorescein diacetate (DCFH-DA; Invitrogen) fluorescent probe, as a previously reported method [21]. In brief, after removing the culture medium, DCFH-DA (10 μM), diluted in a serum-free medium, was added to the cells and incubated for 30 min at 37 °C. Next, the cells were washed three times with phosphate-buffered saline (PBS). The cells were incubated in PBS, and fluorescence intensity was measured by a fluorescence microplate reader (ex. 488 nm/em. 535) (TECAN). The level of total intracellular ROS, paralleled by an increase in fluorescence intensity, was calculated as the percentage of control cells. 

### 2.4. Measurement of Nitrite 

Confluent cells grown on 6-well plates were treated with or without AE-GBE in the presence of LPS (1 μg mL^−1^) for 48 h. Nitrite contents from extracellular were performed using a Griess reagent of 1% sulphanilamide and 0.1% N-(1-naphthyl)-ethylenediamine dihydrochloride in 2.5% phosphoric acid. After reacting at room temperature for 10 min, measurements were made at 540 nm using sodium nitrite as a standard material. 

### 2.5. Measurement of Transepithelial Electrical Resistance 

Transepithelial electrical resistance (TEER) is a well-known quantitative technique for measuring the tight junction integrity in cell culture models of endothelial and epithelial monolayers. The Caco-2 cells were seeded on a Transwell insert plate (Corning Costar Corp., Cambridge, MA, USA) at a density of 1.0 × 10^5^ cells. Cells were co-cultured for 0, 2, 4, 6 and 8 h, with or without LPS (1 μg mL^−1^) with AE-GBE. The TEER values were measured using an ohmmeter with chopstick electrodes (EVOM2, World Precision Instruments, Sarasota, FL, USA) [22]. Before analysing, electrodes were sterilized and equilibrated in deionized water. Measurements were performed within 5 min outside the incubator to minimize the effect of temperature. The data were presented as unit area resistance calculated by dividing resistance values by the effective membrane area. Inserts without cells were used as blanks.

### 2.6. Measurement of Cytokines by Quantitative Reverse Transcription Polymerase Chain Reaction (Quantitative RT-PCR)

Total RNA was progressed using Trizol reagent (Invitrogen, Grand Island, NY, USA), and the concentration and purity were calculated. cDNA synthesis was prepared with a cDNA synthesis kit (Takara Bio Inc., Shiga, Japan) by taking the equal amount of RNA.

qRT-PCR was operated on an ABI real-time PCR system from applied biosystem Inc. (Forster City, CA), using an SYBR Premix Ex Taq II, ROX plus (Takara Bio Inc., Shiga, Japan), as directed by the producer’s manuals. The amplification conditions were proceeded for a total of 40 cycles at 90 °C for 10 min, 95 °C for 15 sec, and 60 °C for 1 min. Gene-specific primers are listed in Table 1. Cyclophilin was used as a reference gene, and all results were normalized to the abundance of cyclophilin mRNA [23]. The relative amounts of mRNAs were calculated with the 2^−ΔΔCt^ method.

### 2.7. Western Blot Analysis

Proteins were extracted with the mammalian protein extraction buffer (Sigma Chemical Co., St. Louis, MO, USA) containing the protease inhibitor cocktail (Sigma) and phenyl methane sulfonyl fluoride (PMSF, Sigma). Protein concentrations in supernatants collected after centrifugation of protein lysates were established using protein assay dye reagent concentrates (Bio-Rad Laboratories, Hercules, CA, USA). Equal amounts of protein were split by SDS-PAGE and transferred onto a nitrocellulose membrane (Amersharm, GE Healthcare Life science, Germany). Primary antibodies were reacted with anti-occludin (1:1000; Invitrogen, USA), anti-claudin-1 (1:1000; Invitrogen), anti-ZO-1 (1:1000; Invitrogen), iNOS (1:500; Santa cruz, USA), COX2 (1:1000; Cell signalling), 4-hydroxynonenal (4HNE) (1:1000; Abcam, UK), AMPK (1:1000; Cell signalling), phospho-AMPK (1:1000; Cell signalling), and anti-β-actin (1:2500; Abcam, Cambridge, UK) at 4 °C overnight, respectively. After secondary antibody reaction for 2 h at room temperature, target protein bands were visualized by an enhanced chemiluminescence method (ECL, Millipore, Boston, MA, USA). Quantity of luminescence was performed using Quantity 1 version 4.6.7 software (Bio-Rad Laboratories, Hercules, CA, USA). 

### 2.8. Measurement of Cytokines by Enzyme Linked Immunosorbent Assay (ELISA)

To examine released cytokines, Caco-2 cells were seeded into 24-well plates at a concentration of 5.0 × 10^4^ cells/well for 24 h. Then, cells were treated with AE-GBE with or without LPS (1 μg mL^−1^) for 24 h and supernatants were used to determine the levels of TNFα, IL-1β, and IL-6 using Duoset ELISA kit (R&D systems, Minneapolis, MN, USA), according to the manufacturer’s instructions.

### 2.9. Statistical Analysis

All results are marked as mean ± standard deviation (SD). Statistical analysis was practiced by SPSS 20.0 software (IBM SPSS version 20.0.0 for Windows, IBM Co., Armonk, NY, USA), and the LSD comparison test was applied to verify the significance of differences between groups. Statistical significance was set at *p* < 0.05.

## 3. Results

### 3.1. Treatment with AE-GBE Attenuates LPS-Induced Cytotoxicity in Caco-2 Cells

**Figure 1:**
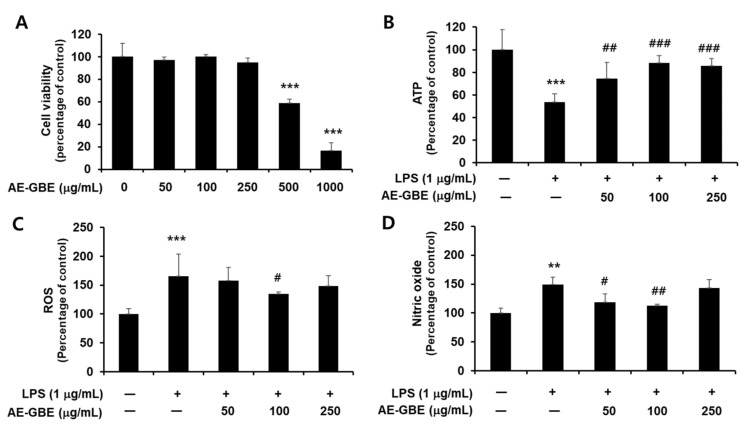
Cytotoxicity of AE-GBE and cytoprotective effect through inhibition of nitrite and ROS in LPS-induced Caco-2 cells. (**A**) Cytotoxicity to dose response of AE-GBE using MTT assay. (**B**) Dose-specific cytoprotective effect of AE-GBE on LPS-induced cell viability using ATP assay. (**C**) Dose-effects of AE-GBE on LPS–induced ROS production in cells. (**D**) Dose-effects of AE-GBE on nitric oxide (NO) level in LPS–treated cells. Values are expressed as percentage of negative control (CON). Results are shown as mean ± SD (*n* = 3–5). **: *p* < 0.01 and ***: *p* < 0.001 versus negative control. #: *p* < 0.05, ##: *p* < 0.01, and ###: *p* < 0.001 versus LPS treated only.

We first confirmed the cytotoxicity of AE-GBE to Caco-2 cells and determined that Caco-2 cells exhibited signs of cytotoxicity at a concentration of 0.5 mg/mL AE-GBE, compared with the untreated control (*p* < 0.001; Figure 1A). We then determined whether nontoxic AE-GBE concentrations protected Caco-2 cells against LPS-induced cytotoxicity by analysing intracellular ATP levels. We found that LPS-induced Caco-2 cells experienced a 46% decrease in intracellular ATP levels, compared with the control group (Figure 1B). However, treatment with AE-GBE at doses of 50, 100, and 250 μg/mL significantly increased the ATP levels of cells that were co-treated with LPS, compared with the LPS-only group. LPS promotes reactive oxygen species (ROS) formation under the inflammatory response, and high nitric oxide (NO) concentrations in the intestine can trigger inflammatory reactions [24]. Thus, we investigated the inhibitory effects of AE-GBE on ROS and NO increased by LPS in Caco-2 cells. LPS alone significantly increased the Caco-2 cell intracellular ROS levels (*p* < 0.001), whereas these were significantly reduced by AE-GBE at a dose of 100 μg/mL (*p* < 0.05; Figure 1C). Moreover, the LPS-only groups significantly increased NO production in Caco-2 cells by approximately 1.5-fold (*p* < 0.01), but these levels were significantly reduced at concentrations of 50 μg/mL (*p* < 0.05) and 100 μg/mL (*p* < 0.01) (Figure 1D).

### 3.2. Treatment with AE-GBE Attenuates LPS-Induced Inflammatory Mediators in Caco-2 Cells

**Figure 2:**
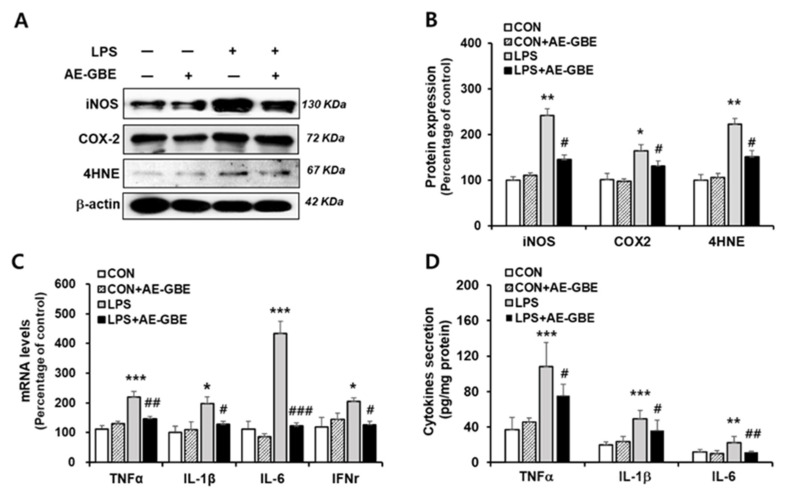
Effects of AE-GBE on inflammatory mediators in LPS-induced Caco-2 cells. iNOS, COX-2 and 4HNE protein expressions in Caco-2 cells were determined via (**A**) Western blotting and (**B**) quantified. (**C**) TNFα, IL-1β, IL-6, and IFNγ expressions were analysed using qRT-PCR. (**D**) TNFα, IL-1β, and IL-6 proteins in culture medium were detected by ELISA assay. Data represent mean ± SD (*n* = 3–5). *: *p* < 0.05, **: *p* < 0.01, and ***: *p* < 0.001versus the CON group; #: *p* < 0.05, ##: *p* < 0.01 and ###: *p* < 0.001 versus the LPS alone group.

After determining 100 μg/mL of AE-GBE to be an effective concentration, we examined the anti-inflammatory effect of LPS treatment with or without 100 μg/mL AE-GBE. The iNOS and COX-2 expression levels increased by more than 2.4-fold (*p* < 0.01) and 1.6-fold (*p* < 0.05), respectively, compared with the control. However, treatment with 100 µg/mL AE-GBE significantly downregulated the expression of COX-2 (*p* < 0.05) and iNOS (*p* < 0.05) compared with the LPS-only groups. The ability of AE-GBE to inhibit lipid peroxidation produced by LPS treatment was determined by the lipid peroxide value marker 4HNE. We found that LPS increased 4HNE expression by 2.2-fold (*p* < 0.01), and that LPS-mediated lipid peroxidation was effectively inhibited by AE-GBE (Figure 2A,B). Abnormal elevation of inflammatory cytokines TNF-α, IL-1β, IL-6 have been observed in IBD [25]. Therefore, we confirmed AE-GBE’s inhibition of the inflammatory response in intestinal epithelial cells by measuring the expression and secretion levels of these inflammatory factors by quantitative RT-PCR and ELISA assays. As indicated in Figure 2C, Caco-2 cells treated with LPS had significantly increased mRNA levels of TNF-α, IL-1β, IL-6, and IFNγ, whereas these expressions were significantly decreased when the cells were treated with 100 μg/mL AE-GBE. Protein levels of TNF-α, IL-1β and IL-6 were also increased by LPS treatment, and it significantly reduced AE-GBE-treated cells with LPS treatment (Figure 2D).

### 3.3. Treatment with AE-GBE Attenuates Transepithelial Electrical Resistance (TEER) in LPS-Treated Caco-2 Cells

**Figure 3:**
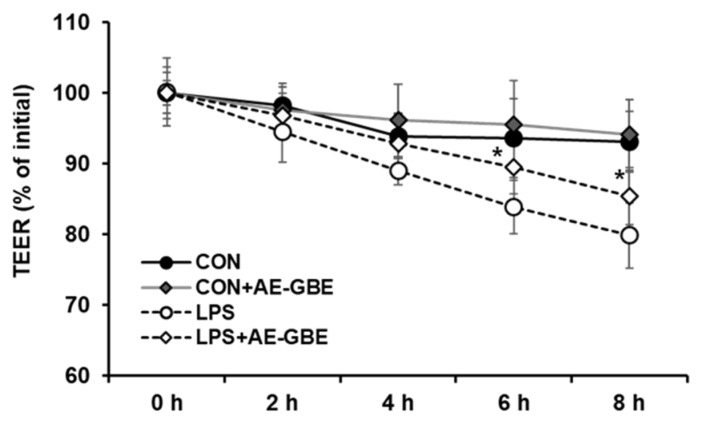
Protective effects of AE-GBE on permeabilization of Caco-2 cell measured by transepithelial electrical resistance method. Levels of TEER were assessed at 2, 4, 6, and 8 h after LPS or ADLE treatment. The data represent mean ± S.D. *: *p* < 0.05 compared with the LPS alone group, *n* = 3–5.

LPS is a large glycolipid composed of lipids and polysaccharides and is applied to induce intestinal barrier permeability [26,27]. We evaluated TEER values to investigate the potential protective effects of AE-GBE on Caco-2 cell monolayer barrier function. After 8 h of LPS treatment, the TEER value of the Caco-2 monolayer was about 20% lower than that of the control group. However, 6 h after treating the Caco-2 monolayer with LPS in the presence of AE-GBE, the TEER increased significantly compared with LPS alone. Moreover, the TEER of the Caco-2 monolayer did not change after treatment with AE-GBE alone (Figure 3).

### 3.4. Treatment with AE-GBE Activates AMPK and Enhances the TJ Protein Expression Level in LPS-Treated Caco-2 Cells

**Figure 4:**
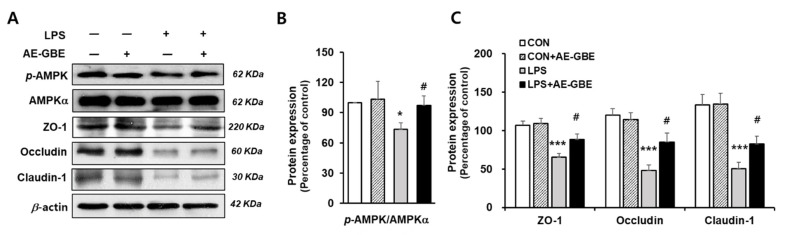
Effects of AE-GBE on AMPK activation and TJ-related proteins in LPS-induced Caco-2 cells. AMPKα, *p*-AMPK, and tight-junction protein (ZO-1, occludin, and claudin-1) expressions in Caco-2 cells were determined via Western blotting (**A**) and quantified (**B**,**C**). The data in the figures represent mean ± S.D. *: *p* < 0.05 and ***: *p* < 0.001 versus the CON group; #: *p* < 0.05 compared with the LPS alone group, *n* = 3–5.

The AMPK activation signalling pathway is critical in intensifying the epithelial apical junction and protecting the epithelial barrier from environmental stress [28,29]. We investigated whether AE-GBE changed the AMPK activity and its associated TJ expression in LPS-induced Caco-2 cells. We found that LPS treatment significantly inhibited AMPK activation by about 27% (*p* < 0.05), and that these levels were restored to the control level following AE-GBE treatment (*p* < 0.05; Figure 4A,B). LPS also decreased the Caco-2 cell expression of ZO-1, occludin, and claudin-1 to 30%, 41%, and 38% of the control, respectively (*p* < 0.001). AE-GBE significantly preserved TJ protein expression, whereas the TJ protein expression was diminished by LPS (Figure 4C).

## 4. Discussion

The exact aetiology of IBD is unclear, but it has been known to occur due to an abnormality in the gastrointestinal mucosa’s cell-mediated immune response. Invasion by intestinal pathogens has been observed to induce the expression of pro-inflammatory factors in intestinal epithelial cells [30,31,32,33]. Therefore, preventing pro-inflammatory factors is one of the most important therapeutic targets for reducing IBD and its common symptoms, such as severe diarrhoea, fever, and bloody stool.

Several studies have proposed natural IBD treatment products with the ability to reduce the levels of proinflammatory cytokines or mediators. Recently, edible insects have shown anti-inflammatory effects against various disease models [34]. Similarly, AE-GBE is derived from a food source that plays a strong economic role and has also shown significant anti-inflammatory and anti-oxidative stress effects [19,35].

We first confirmed AE-GBE’s cytoprotective effect in LPS-induced Caco-2 cells by reducing the NO content and intracellular ROS. NO overproduction via iNOS upregulation by the intestinal epithelium has been consistently associated with IBD [36]. ROS also play an important role in initiating and perpetuating inflammatory cascades, and antioxidants have been frequently found to attenuate inflammation. The mitochondrial respiratory chain produces NO, and thus reactive nitrogen species that induce excessive lipid peroxidation of other reactive species, including malondialdehyde and 4HNE [37,38]. Sun et al. (2019) demonstrated that 4HNE linked to the inflammatory response was increased in LPS-stimulated microglial cells through the cPLA2/ARA pathway [39]. It was similarly reported that 4HNE–protein adduct levels increased in TNFα-treated Caco-2 cells and were prevented by epicatechin and apocynin treatment [40]. We also found that 4HNE and inflammatory mediators such as iNOS and COX2 were increased in LPS-treated cells and were attenuated by AE-GBE, suggesting that AE-GBE’s anti-inflammatory effect is one of the intestinal protective mechanisms of LPS-treated cells. Moreover, our data suggest the use of edible insect AE-GBE to prevent and/or treat IBD is an effective strategy consistent with previous results regarding the use of antioxidants.

We found that AE-GBE protected against LPS-induced intestinal barrier dysfunction. Accumulating evidence suggests that LPS induces hyperpermeability and interferes with intestinal barrier function [41] by altering TJ protein expression, which leads to increased endotoxin release into portal circulation. The TEER measurement to assess the electrical resistance of the Caco-2 cell monolayer is an indicator of TJ integrity, with higher TEER values implying greater TJ integrity [42]. In this study, we found that alterations of representative TJ proteins in the intestine were induced by AE-GBE treatment. Moreover, AE-GBE decreased intestinal cytokines, as shown by reduced TNF-α, IL-1β, IL-6, and IFN-γ; this may be important in explaining the protective effects of AE-GBE against intestinal mucosal damage.

AMPK, an intracellular energy sensor, benefits gut health by increasing nutrient absorption, reducing gut inflammation, and improving gut barrier function [43]. In particular, AMPK phosphorylation is reported to be involved in TJ assembly in kidney cells [44,45], and AMPK promotes Cdx2 expression to enhance intestinal barrier function and epithelial differentiation [46]. Recently, we obtained results consistent with previous reports on AMPK activation by using an *Allomyrina dichotama* larval extract treatment in LPS-treated Caco-2 cells [22]. In this study, we found that the increment of AMPK phosphorylation by AE-GBE suggested an increase in TJ integrity from LPS-induced disassembly.

Although we did not observe the active ingredients of AE-GBE, Ahn et al. found that glycosaminoglycans isolated from crickets had anti-inflammatory effects in an adjuvant-induced chronic arthritis rat model, and antioxidant activity in liver tissue [19,47]. As AE-GBE inhibited LPS-induced inflammatory factors such as ROS, NO, and cytokines, it can be assumed that biologically active ingredients such as antioxidants have intrinsic activity. As such, research focusing on the isolation of bioactive ingredients with antioxidant effects is currently underway. We found that polar fraction showed high polyphenol content as well as high antioxidant activity (data not shown). Previous reports demonstrated that insects have an anti-oxidant effect as they include antioxidant enzymes, superoxide dismutase [48]. Therefore, the reduction in oxidative stress can be one of the mechanisms of anti-inflammatory effect. 

## 5. Conclusions

In conclusion, we demonstrated that AE-GBE considerably improved epithelial barrier dysfunction by enhancing TJ proteins through reducing the inflammatory response and activating AMPK in intestinal epithelial cells. Together, these results may contribute to the establishment of the pharmacological function of AE-GBE, with the aim of potentially preventing inflammation-related intestinal dysfunction. Thus, these results suggest that AE-GBE can prevent TJ-related proteins, which may enhance intestinal epithelial barrier function by activating the phosphorylation of AMPK, a therapeutic target in intestinal diseases.

## Figures and Tables

**Table 1:** Primer sequences for real-time PCR.

Gene	Forward (5′-3′)	Reverse (5′-3′)
TNF-α	5′-TGCTCCTCACCCACACCAT-3′	5′-GGAGGTTGACCTTGGTCTGGTA-3′
IL-6	5′-GCTGCAGGCACAGAACCA-3′	5′-TAAAG TGCGCAGAATGAGATG-3′
IL-1β	5′-ACGATGCACCTGTACGATCACT-3′	5′-CACCAAGCTTTTTTGCTGTGAGT-3′
IFN*γ*	5′-ACTCATCCAAGTGATGGCTGAA-3′	5′-TCCTTTTTCGCTTCCCTGTTT-3′
Cyclophilin	5′-TGCCATCGCCAAGGAGTAG-3′	5′-TGCACAGACGGTCACTCAAA-3′

## Data Availability

Not applicable.
